# A Novel Pathway for the Biosynthesis of Heme in *Archaea*: Genome-Based Bioinformatic Predictions and Experimental Evidence

**DOI:** 10.1155/2010/175050

**Published:** 2010-12-13

**Authors:** Sonja Storbeck, Sarah Rolfes, Evelyne Raux-Deery, Martin J. Warren, Dieter Jahn, Gunhild Layer

**Affiliations:** ^1^Institute for Microbiology, Technical University of Braunschweig, Spielmannstraße 7, 38106 Braunschweig, Germany; ^2^School of Biosciences, University of Kent, Canterbury, Kent CT2 7NJ, UK

## Abstract

Heme is an essential prosthetic group for many proteins involved in fundamental biological processes in all three domains of life. In *Eukaryota* and *Bacteria* heme is formed *via* a conserved and well-studied biosynthetic pathway. Surprisingly, in *Archaea* heme biosynthesis proceeds *via* an alternative route which is poorly understood. In order to formulate a working hypothesis for this novel pathway, we searched 59 completely sequenced archaeal genomes for the presence of gene clusters consisting of established heme biosynthetic genes and colocalized conserved candidate genes. Within the majority of archaeal genomes it was possible to identify such heme biosynthesis gene clusters. From this analysis we have been able to identify several novel heme biosynthesis genes that are restricted to archaea. Intriguingly, several of the encoded proteins display similarity to enzymes involved in heme *d*_1_ biosynthesis. To initiate an experimental verification of our proposals two *Methanosarcina barkeri* proteins predicted to catalyze the initial steps of archaeal heme biosynthesis were recombinantly produced, purified, and their predicted enzymatic functions verified.

## 1. Introduction

**Figure 1:**
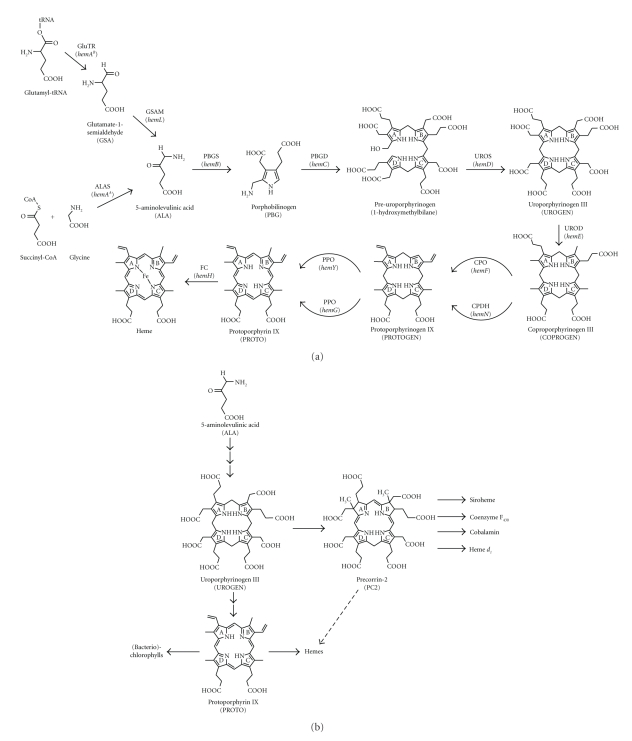
Tetrapyrrole biosynthesis pathways. (a) Heme biosynthesis in most bacteria and the *Eukaryota*. The first common precursor in the classical heme biosynthesis pathway is ALA of which eight molecules are converted into UROGEN in three consecutive enzymatic steps. UROGEN is then further converted into heme through successive modifications of the macrocycle side chains and finally iron insertion. The enzymes involved in the classical heme biosynthesis are glutamyl-tRNA reductase (GluTR), glutamate-1-semialdehyde-2,1-aminomutase (GSAM), 5-aminolevulinic acid synthase (ALAS), porphobilinogen synthase (PBGS), porphobilinogen deaminase (PBGD), uroporphyrinogen III synthase (UROS), uroporphyrinogen III decarboxylase (UROD), oxygen-dependent coproporphyrinogen III oxidase (CPO), coproporphyrinogen III dehydrogenase (CPDH), oxygen-dependent and oxygen-independent protoporphyrinogen IX oxidase (PPO), and ferrochelatase (FC). The corresponding bacterial gene names are denoted in brackets below the enzyme names. (b) Overview of the different branches of the tetrapyrrole biosynthesis pathway. The last common precursor for the formation of all tetrapyrroles is UROGEN. Hemes and (bacterio)chlorophylls share PROTO as their last common intermediate. Siroheme, cobalamin, coenzyme F_430_, and heme *d*_1_ are all biosynthesized *via* precorrin-2. In the *Archaea* and some bacteria an alternative heme biosynthesis pathway exists in which the heme is biosynthesized from precorrin-2 *via* as yet unknown intermediates.

Heme, a modified tetrapyrrole, acts as an essential prosthetic group in many enzymes, sensory, and regulatory proteins. Hemes are also essential components of electron transport chains driving aerobic and anaerobic respiration and photosynthesis in almost all living organisms. Consequently, heme-containing proteins are found in all three domains of life, the *Eukaryota*, the *Bacteria*, and the *Archaea*. The biosynthesis of this important and ubiquitously distributed molecule has been intensively studied in eukaryotic and bacterial organisms, but little is known about heme biosynthesis in archaea. It is now well established for bacteria and eukarya that heme biosynthesis proceeds along a conserved pathway with highly related enzymes and identical biosynthetic intermediates (Figure 1(a)) [1]. Heme synthesis represents just one component of a larger, branched tetrapyrrole biosynthesis pathway, which is also responsible for the synthesis of chlorophylls, bacteriochlorophylls, cobalamin, siroheme, heme *d*_1_ and coenzyme F_430_ (Figure 1(b)) [2]. 

The common precursor for the formation of heme and all other tetrapyrroles is 5-aminolevulinic acid (ALA). Depending on the organism this molecule is either synthesized through the condensation of glycine and succinyl-CoA (Shemin pathway) by ALA synthase (HemA^A^) or in a two-step enzymatic process from glutamyl-tRNA *via* the intermediate glutamate-1-semialdehyde (GSA) by glutamyl-tRNA reductase (HemA^B^) and GSA-2,1-aminomutase (HemL) (C_5_-pathway) [3, 4]. Eight molecules of ALA are then converted into uroporphyrinogen III (UROGEN), the first cyclic tetrapyrrole of the pathway, in three consecutive enzymatic steps. First, two ALA molecules are condensed by porphobilinogen synthase (HemB) to the pyrrole derivative porphobilinogen (PBG) [5]. In the next step, four PBG molecules are oligomerized to the linear tetrapyrrole pre-uroporphyrinogen by PBG deaminase (HemC) and finally uroporphyrinogen III (UROGEN) is formed by cyclization of the pre-uroporphyrinogen by UROGEN synthase (HemD) [6]. The intermediate uroporphyrinogen III represents the last common precursor for all tetrapyrroles and is therefore an important branchpoint of the pathway. One of the diverting biosynthetic routes leads to the formation of hemes and (bacterio)chlorophylls *via* the intermediate coproporphyrinogen III (COPROGEN) and the other represents the first step of cobalamin, siroheme, heme *d*_1_, and coenzyme F_430_ biosyntheses *via* the common intermediate precorrin-2. COPROGEN and precorrin-2 are formed from UROGEN by the key branchpoint enzymes uroporphyrinogen III decarboxylase (HemE) and *S*-adenosyl-L-methionine-dependent uroporphyrinogen III methyltransferase (SUMT), respectively. Eukaryotic and bacterial heme biosynthesis further proceeds *via* the conversion of COPROGEN into protoporphyrinogen IX (PROTOGEN) by coproporphyrinogen III oxidase (HemF) or dehydrogenase (HemN) and the subsequent oxidation by protoporphyrinogen IX oxidase (HemY, HemG) to protoporphyrin IX (PROTO) [7]. Finally, the insertion of ferrous iron into PROTO by ferrochelatase (HemH) yields the end product heme [8]. All heme biosynthetic enzymes have been purified from many different eukaryotic and bacterial organisms and biochemically characterized [1]. The corresponding genes (*hemA*, *L*, *B*, *C*, *D*, *E*, *F*, *N*, *Y*, *G*, *H*) have all been cloned and sequenced [9]. In Figure 1(a) the commonly used gene designations for all bacterial heme biosynthesis genes are given together with their corresponding enzyme names.

In two independent bioinformatics studies in 2002 and 2008 the distribution of heme biosynthetic genes in prokaryotic organisms was investigated by analysis of the currently available sequenced microbial genomes [10, 11]. It was found that almost all bacteria which synthesize heme *de novo* possess the complete set of *hem* genes (i.e., *hemA^A^* or *hemA^B^*, *hemL*, *hemB*, *hemC*, *hemD*, *hemE*, *hemF* and/or *hemN*, *hemY* or *hemG*, *hemH*). In contrast, some bacteria (e.g., *Clostridia* and *Desulfovibrio* species) and almost all archaea were found to possess only the genes encoding the enzymes required for UROGEN formation (*hemA^B^*, *L*, *B*, *C*, *D*) and lacked the genes encoding the enzymes necessary for the conversion of UROGEN into heme [10, 11]. Such a finding can be explained by (i) the possibility that these organisms have no need for heme and require the initial genes for cobalamin, siroheme, coenzyme F_430_, or heme *d*_1_ formation, (ii) the possibility that they take up heme from the environment, or (iii) the existence of an alternative, yet unknown, heme biosynthesis pathway. For many free-living bacteria and archaea heme uptake is not very likely since heme is simply not available in their environment. However, a few examples of such cases exist in archaea and bacteria [12, 13]. Moreover, several pathogenic bacteria take up heme from their host and use it as an iron source. However, these bacteria often possess an intact heme biosynthetic apparatus [11]. 

It has been known for some time that *Desulfovibrio* species and many archaea contain cytochromes and other heme-containing proteins [14–22], and therefore they must be able to synthesize their own heme. Indeed, for the sulfate-reducing bacterium *Desulfovibrio vulgaris* and the methanogenic archaeon *Methanosarcina barkeri* it was shown experimentally that an alternative heme biosynthesis pathway must exist. In these cases *in vivo* labeling studies demonstrated that their hemes contain methyl groups on rings A and B that are derived from methionine (*via S*-adenosyl-L-methionine) and not from ALA as is the case for hemes synthesized *via* the classical pathway [23, 24]. Further, in *D. vulgaris* sirohydrochlorin (the oxidized form of precorrin-2), 12,18-didecarboxysirohydrochlorin, coproporphyrin III, and PROTO were isolated as potential heme biosynthesis intermediates [25]. Thus, the alternative heme biosynthesis pathway seems to branch off the classical pathway at the stage of UROGEN. In the first step of the alternative route UROGEN is methylated at rings A and B by a SUMT-like enzyme to yield precorrin-2 (Figure 1(b)). This SUMT-dependent methylation of UROGEN is also required for the biosyntheses of cobalamin, siroheme, heme *d*_1_, and coenzyme F_430_ (Figure 1(b)). Recently, in *D. vulgaris* a bifunctional enzyme carrying both UROGEN synthase and SUMT activities and a precorrin-2 dehydrogenase (PC2-DH) catalyzing precorrin-2 oxidation to sirohydrochlorin were biochemically characterized [26]. Both enzymes are probably involved in the alternative heme biosynthesis pathway in this organism.

Besides the *in vivo* labeling study in *M. barkeri* the alternative heme biosynthesis pathway has not been investigated in archaea, so far. In the last few years the number of completely sequenced archaeal genomes has greatly increased and therefore we decided to start our investigation of archaeal heme biosynthesis with the search for potential heme biosynthesis gene clusters within these genomes. We found that many archaea indeed contain gene clusters consisting of the known early heme biosynthesis genes (*hemA^B^*, *hemL*, *hemB*, *hemC*, *hemD*) and of “*nir*-like” genes which encode proteins homologous to proteins involved in heme *d*_1_ biosynthesis in denitrifying bacteria. Moreover, very often the genes encoding a putative SUMT and a potential PC2-DH were found localized in these archaeal heme biosynthesis gene clusters. Here, the predicted SUMT and PC2-DH from *M. barkeri* were recombinantly produced, purified and shown *in vitro* to carry SUMT and PC2-DH activity, respectively. 

## 2. Materials and Methods

### 2.1. Chemicals

All chemicals, reagents, and antibiotics were obtained from Sigma-Aldrich (Taufkirchen, Germany) or Merck (Darmstadt, Germany). DNA polymerase, restriction endonucleases, and PCR requisites were purchased from New England Biolabs (Frankfurt a.M., Germany). Oligonucleotide primers were obtained from *meta*bion international AG (Martinsried, Germany). PCR purification and gel extraction Kits were purchased from Qiagen GmbH (Hilden, Germany). Ni Sepharose 6 Fast Flow was purchased from GE Healthcare (München, Germany). Uroporphyrin III was obtained from Frontier Scientific Europe (Carnforth, UK).

### 2.2. Construction of Vectors for Recombinant Protein Production

The gene *mba_A1461* encoding a potential PC2-DH from *M. barkeri* was PCR amplified using the primers 01CysGN_Mba_*Bam*HI_fw (GAA GGG ATC CGA TGA CCA AAA CCA ATA ATT TTC) and 02CysGN_Mba_*Not*I_rev (GAA CGC GGC CGC TTA ACG GTT GCT GTT CAC) containing *Bam*HI and *Not*I restriction sites (underlined) and cloned into appropriately cut pET-Duet-1 (Novagen, Darmstadt, Germany) to generate pET-Duet_*mba_A1461*. The plasmid pMA_*mba_A1461* (GeneART, Regensburg, Germany), which contains a synthetic copy of the *mba_A1461* gene, codon-optimized for expression in *Escherichia coli*, was used as the DNA template for PCR. 

The gene *mba_A1791* encoding a putative SUMT from *M. barkeri* was PCR amplified using the primers MbarcobA-ATG (CAC ATA TGT CAG GAA ATT ACG GAA AAG) and MbarcobA-Stop (AGG ATC CAA AAC TAG TTA AAA GTC AAC TCC TGT CCG) containing *Nde*I and *Spe*I-*Bam*HI restriction sites (underlined) from genomic *M. barkeri* DNA. The resulting PCR fragments and the vector pET14b (Novagen) were subsequently digested with *Nde*I and *Bam*HI and ligated to generate pET14b_*mba_A1791*.

### 2.3. Bacterial Strains and Growth Conditions

*E. coli* DH10B was used as the host for cloning. For production of recombinant proteins the *E. coli* strains BL21 (DE3) and BL21 Star (DE3) pLysS were used, respectively. The expression vector pET14b_*mba_A1791* was transformed into *E. coli* BL21 Star (DE3) pLysS. The vector pET-Duet_*mba_A1461* was transformed into *E. coli* BL21 (DE3). For recombinant protein production the *E. coli* strains carrying the corresponding vectors were grown at 37°C in LB-medium containing appropriate antibiotics. Protein production was induced by adding 50 *μ*M isopropyl isopropyl-*β*-D-thiogalactopyranosid (IPTG) to the cultures at an optical density at 578 nm of 0.6. The *E. coli* BL21 (DE3) strain containing pET-Duet_*mba_A1461* was further cultivated at 37°C for 4 h. The *E. coli* BL21 Star (DE3) pLysS strain containing pET14b_*mba_A1791* was further cultivated at 17°C for 18 h. The cells were harvested by centrifugation and stored at −20°C.

### 2.4. Purification of Enzymes and Tetrapyrroles

The affinity chromatographic purification of the recombinant enzymes was performed as described before [27] with minor changes. Briefly, for resuspension of the *E. coli* cells, harbouring the produced recombinant protein, buffer A (50 mM Tris/HCl (pH 7.5), 300 mM NaCl, 10% (w/v) glycerol) was used containing 1 mM phenylmethanesulfonyl fluoride. The cells were disrupted using a French press (1000 p.s.i.) and the soluble protein fraction was obtained by ultracentrifugation (60 min, 175000 × g, 4°C). The supernatant was applied to 1 mL of Ni Sepharose 6 Fast Flow (GE Healthcare). The flow-through containing the tetrapyrroles accumulated during *in vivo* protein production was applied to a 1 mL silica gel 100 C_18_-reversed phase column (Sigma-Aldrich) and the tetrapyrroles were extracted as described before [27]. The Ni-resin with bound proteins was washed extensively with buffer A. After a preelution step with buffer A containing 20 mM imidazole the recombinant protein was eluted with buffer A containing 300 mM imidazole. Immediately after elution a buffer exchange was performed in an anaerobic chamber (Coy Laboratories, Grass Lake, MI, USA) by passing the protein solution through a NAP-25 column (GE Healthcare) that had been equilibrated with degassed buffer A containing 5 mM dithiothreitol. The protein was stored at −20°C until required.

### 2.5. Determination of Protein Concentration

The Bradford Reagent (Sigma-Aldrich) was used to determine protein concentrations, according to the manufacturer's instructions, using BSA as a standard.

### 2.6. Molecular Mass Determination

In order to determine the oligomeric state of proteins a gel permeation chromatography was performed using a Superdex 200 10/30 GL column with an ÄKTA Purifier system (GE Healthcare). The column was equilibrated with buffer A containing 5 mM dithiothreitol and calibrated using four standard proteins: cytochrome c, conalbumin, alcohol dehydrogenase, *β*-amylase (Kit Gel Filtration molecular weight markers (Sigma-Aldrich)). Protein samples (1 mg mL^−1^) were applied to the column and the elution of proteins was monitored by determination of the absorption of the eluate at 280 nm as described before [27].

### 2.7. In Vitro Enzyme Activity Assays

*In vitro* enzyme activities of the recombinantly produced and purified Mba_A1791 and Mba_A1461 proteins were measured using a coupled enzyme assay as described before [27]. The assay was performed in an anaerobic chamber (Coy Laboratories) under strictly anaerobic conditions (O_2_ = 0 ppm). The substrate uroporphyrinogen III was generated enzymatically from 1 mM ALA using purified HemB (0.14 *μ*M) from *Pseudomonas aeruginosa*, HemC (0.15 *μ*M), and HemD (0.17 *μ*M) both from *Bacillus megaterium* in a final volume of 1 mL of degassed buffer B containing 50 mM Tris/HCl (pH 8.0), 100 mM KCl, 5 mM MgCl_2_, and 50 mM NaCl. In order to investigate the activity of Mba_A1791 the enzyme was added to a final concentration of 1.5 *μ*M, and SAM as methyl donor was added to a final concentration of 200 *μ*M. In order to determine the activity of Mba_1461, precorrin-2 was generated using *P. aeruginosa* NirE as SUMT [27] at a concentration of 1.5 *μ*M. The Mba_1461 was added to a final concentration of 1.5 *μ*M with 100 *μ*M NAD^+^. The reaction mixtures were incubated overnight at 37°C in the dark. UV-visible spectra of the assay mixtures were recorded on a V-650 spectrophotometer (Jasco, Gross-Umstadt, Germany).

### 2.8. Bioinformatics Analysis

For the analysis and comparison of archaeal genomes the “Microbial Genome Database for Comparative Analysis” (http://mbgd.genome.ad.jp/) was used [28–30]. This database contains a total of 68 completely sequenced archaeal genomes. Of these 68 genomes we initially chose one for each species analyzed, that is, different strains within one species were not included, which left us with 59 genomes. The archaeal species whose genomes were chosen are listed in Table 1. We also included the genomes of *E. coli*, *P. aeruginosa*, and *D. vulgaris* as positive and negative controls for our search. First, the database was searched for the known early heme biosynthesis genes of *E. coli* (*hemA^B^*, *hemL*, *hemB*, *hemC*, *hemD*) and then the database was used to find “orthologous clusters” in the archaeal genomes. With the “orthologous cluster” tool all homologous *hem* genes in the chosen genomes were displayed and a multiple genome map comparison could be viewed. For the clustering parameters we chose the default values of the database. Using the “multiple genome map comparison” tool we identified gene clusters with similar gene organization in the neighborhood of the known early heme biosynthesis genes in the archaeal genomes.

## 3. Results and Discussion

### 3.1. The Late Heme Biosynthesis Genes Are Missing in Archaeal Genomes

In order to identify potential heme biosynthesis gene clusters in the 59 archaeal genomes analyzed we first checked for the presence and genomic localization of the early heme biosynthesis genes *hemA^B^*, *hemL*, *hemB*, *hemC*, and *hemD.* Next, we inspected the genes located in their direct neighborhood using the MBGD database. Out of the 59 archaeal genomes included in this study we found 12 genomes which do not contain any obvious *hem* gene (Table 1). These organisms apparently do not synthesize tetrapyrroles *de novo* unless *via* a completely novel pathway. Alternatively, these members of the *Archaea* do not need heme and other tetrapyrroles, respectively, or they are able to take up these compounds from their environment as described previously [12]. For example, it was reported recently that many archaeal species possess genes that encode putative homologs of the prokaryotic BtuFCD system for cobalamin uptake [31]. Here, in the 12 genomes that lack the *hem* genes we also found *btuFCD* homologs with the exception of the *Korarchaeum cryptofilum* and the *Nanoarchaeum equitans* genomes (not shown).

However, in the majority (47) of the studied genomes we found all five *hem* genes (*hemA^B^*, *hemL*, *hemB*, *hemC*, *hemD*) whose encoded protein products are known to be responsible for formation of the tetrapyrrole precursor UROGEN. One exception is the genome of *Aeropyrum pernix* which is missing a recognizable *hemD* gene. As already observed before [10, 11] we failed to detect the genes *hemE*, *hemF/N*, *hemG/Y*, *hemH* encoding the known late heme biosynthesis enzymes catalyzing the conversion of UROGEN into heme. Exceptions from this rule came from the analysis of the *Picrophilus torridus*, *Thermoplasma acidophilum*, and *Thermoplasma volcanium *genomes. The genomes of these three species contain *hemE* and *hemH* genes encoding UROGEN decarboxylase and ferrochelatase, respectively, as highlighted in earlier studies [10, 11]. However, no genes encoding recognizable COPROGEN oxidases/dehydrogenases (*hemF/N*) or PROTOGEN oxidases (*hemG/Y*) were found. Thus, most archaea possess the genetic potential for synthesizing UROGEN from glutamyl-tRNA *via* the intermediates GSA, ALA, PBG, and pre-uroporphyrinogen and appear to have genes for heme-containing proteins. Therefore, to make heme they must transform the UROGEN by a novel pathway that differs from the known heme biosynthesis route. This is consistent with the observation that the methanogenic archaeon *M. barkeri* synthesizes its heme *via* the intermediate precorrin-2 [24].

### 3.2. Archaeal Hem Genes Are Clustered with SUMT and PC2-DH Genes

**Figure 2:**
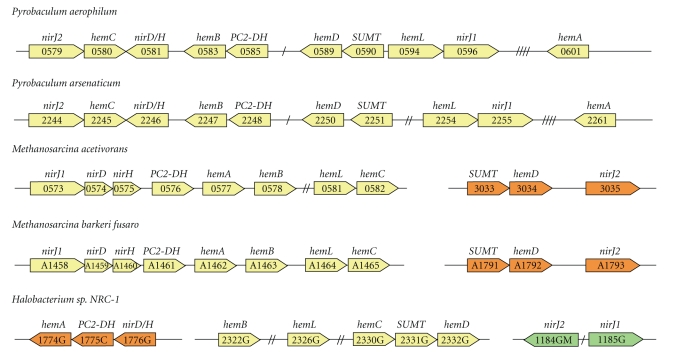
Putative heme biosynthesis gene clusters. The most complete gene clusters containing the *hem*, *SUMT*, *PC2-DH*, and *ahb-nir* genes were found in the genomes of different *Pyrobaculum* species, members of the *Methanosarcinales* and *Halobacteria* (see also Table 1). Genes located as direct or near neighbors are shown in the same color. The number of slashes indicates the number of genes encoding hypothetical proteins which interrupt the gene cluster. The database gene numbers are given in the gene arrow representation without the abbreviation for the strain. These abbreviations are PAE for *P. aerophilum*, PARS_ for *P. arsenaticum*, MA for *M. acetivorans*, MBAR_ for *M. barkeri fusaro*, and VNG for *Halobacterium sp. NRC-1*.

Upon closer bioinformatical inspection of the chromosomal organization of the detected archaeal *hem* genes we found that they are often located within gene clusters comprising two or more *hem* genes (Table 1 and Figure 2). Interestingly, within these *hem* gene clusters we also detected genes potentially encoding a SUMT and a precorrin-2 dehydrogenase (PC2-DH). SUMT proteins catalyze the *S*-adenosyl-L-methionine-dependent methylation of UROGEN on rings A and B at positions 2 and 7 to give precorrin-2 (Figure 1(b)). The PC2-DH proteins in turn oxidize the precorrin-2 to sirohydrochlorin in a NAD^+^-dependent reaction. Unfortunately, the nomenclature for these two genes in the MBGD database is quite inconsistent as the SUMT encoding gene is sometimes named *cobA*, *cysG-1*, *cysG-2*, *cysG*, *uroM*, or *hemX*, and the gene encoding PC2-DH is referred to as *sirC*, *hemX*, *cysG*, or *cysG1*. In the following we will refer to the genes encoding the methyltransferase and the dehydrogenase simply as the *SUMT* and *PC2-DH* genes, respectively. In archaea a SUMT is probably required for the synthesis of all tetrapyrroles including heme, cobalamin, siroheme, and coenzyme F_430_ [24, 32]. The PC2-DH has been shown conclusively to be involved in siroheme and anaerobic cobalamin formation in bacteria [33, 34] and will probably fulfill this function also in archaea. Its involvement in heme and coenzyme F_430_ biosyntheses has not yet been demonstrated and requires further experimental evidence. 

Although both enzymes are required for the formation of all these different tetrapyrroles in archaea, it is interesting to note that almost all archaeal species possess only one *SUMT* and one *PC2-DH* gene. The only exception from this rule is *Archaeoglobus fulgidus* which possesses two *SUMT* genes. As outlined above, the *SUMT* and *PC2-DH* genes are often clustered on the genomes with the early *hem* genes. This clustering of genes encoding the enzymes responsible for the transformation of ALA into precorrin-2 or sirohydrochlorin provides the organisms with the possibility of coordinated gene expression and production of enzymes catalyzing consecutive biosynthetic steps. However, such heme biosynthesis gene clusters were not found in all archaeal genomes. For some of the investigated species, like *Ignicoccus hospitalis* and *Caldivirga maquilingensis*, the heme biosynthesis genes were found scattered randomly throughout the genome (Table 1).

### 3.3. Potential Involvement of *nir*-Like Genes in Archaeal Heme Biosynthesis

Interestingly, in 32 of the archaeal genomes that contain the early *hem* genes we also found so-called *nir* genes (*nirD*, *nirH, nirJ*) co-localized in large gene clusters with *hemA^B^*, *hemL*, *hemB*, *hemC*, *hemD*, *SUMT*, and *PC2-DH* genes (Table 1 and Figure 2). It was previously reported that *D. vulgaris* and some methanogenic archaea harbor these *nir* genes on their genomes. It was speculated that the *nir* genes might be involved in the alternative heme biosynthesis pathway in these organisms [26]. Here, we show not only that the methanogenic archaea contain *nir* genes, but also that the majority of archaea that synthesize heme *de novo* require these genes (Table 1). These *nir* genes encode proteins that are homologous to proteins involved in heme *d*_1_ biosynthesis in denitrifying bacteria such as *P. aeruginosa*. The dioxoisobacteriochlorin heme *d*_1_ serves as an essential prosthetic group in the cytochrome *cd*_1_ nitrite reductase which catalyzes the second step of denitrification [36]. However, based on amino acid sequence homology searches, only *Pyrobaculum aerophilum*, *Pyrobaculum arsenaticum*, and *Pyrobaculum calidifontis* possess a potential cytochrome *cd*_1_ nitrite reductase. All other archaeal genomes analyzed in this study do not. Consequently, the majority of archaeal *nir* genes are not involved in heme *d*_1_ biosynthesis. Rather, they are likely to be involved in heme biosynthesis. Therefore, we renamed these *nir*-like genes in the *Archaea ahb*(archaeal heme biosynthesis)-*nir* genes. 

### 3.4. Structures of Potential Heme Biosynthesis Gene Clusters in Archaea

As mentioned above, the *ahb-nir* genes are often clustered with the *hem*, *SUMT*, and *PC2-DH* genes on the archaeal genomes. The most complete gene clusters, comprising ten out of the eleven potential heme biosynthesis genes, were found in the genomes of *P. aerophilum* and *P. arsenaticum* (Figure 2). In *P. aerophilum* these genes form one large, uninterrupted gene cluster. Another striking clustering of the potential heme biosynthesis genes was observed in the *Methanosarcinales* (Figure 2). For example, in the genomes of *M. acetivorans* and *M. barkeri* the genes *hemA^B^*, *hemL*, *hemB*, *hemC*, *ahb-nirD*, *ahb-nirH*, *ahb-nirJ1*, and *PC2-DH* are organized as one continuous gene cluster, while the genes *hemD*, *ahb-nirJ2*, and *SUMT* are localized together in a second gene cluster. In *Halobacterium sp.* NRC-1 three heme biosynthesis-related gene clusters were found. The first cluster comprises the genes *hemL*, *hemB*, *hemC*, *hemD*, and *SUMT*, the second consists of *hemA^B^*, *PC2-DH*, *ahb-nirD*, and *ahb-nirH* and the third contains *ahb-nirJ1*, and *ahb-nirJ2* (Figure 2). In the other archaeal species that possess *ahb-nir* genes the clustering with the *hem*, *SUMT*, and *PC2-DH* genes is less distinct, but there is still often a colocalization of one or two *ahb-nir* genes with one or several *hem* genes (Table 1).

### 3.5. Proposed Function of the *ahb-nir* Genes during Heme Biosynthesis in the Archaea

As already mentioned above the *ahb-nir* genes encode proteins that are similar to proteins involved in heme *d*_1_ biosynthesis. We compared the amino acid sequences of the Ahb-Nir proteins from *M. barkeri* with the Nir proteins involved in heme *d*_1_ biosynthesis in *P. aeruginosa*. We found the following sequence identities: *M. barkeri* Ahb-NirD and *P. aeruginosa* NirD: 36.3%; Ahb-NirH and NirH: 40.1%; Ahb-NirJ1 and NirJ: 29.5%; Ahb-NirJ2 and NirJ: 38.8%. Thus, the Ahb-NirJ2 is more similar to the heme *d*_1_ biosynthesis protein NirJ than is the Ahb-NirJ1. Additionally, we found an amino acid sequence identity of 31.8% between the two Ahb-NirJ proteins. 

Although the precise functions of the Nir proteins involved in heme *d*_1_ biosynthesis has not yet been established, several reasonable proposals were made [26, 36–40]. First of all, it is known that heme *d*_1_ is biosynthesized from precorrin-2 [27, 35]. In order to obtain heme *d*_1_ from this precursor the following modifications have to take place: (a) decarboxylation of the acetate groups on rings C and D, (b) removal of the propionate side chains on rings A and B and replacement by oxo groups, (c) formation of an acrylate side chain on ring D, (d) oxidation of the tetrapyrrole macrocycle, and (e) iron insertion. The order of these reactions is not known. However, it was proposed that the oxidation reaction (b) might be catalyzed by the NirJ protein during heme *d*_1_ formation [37]. NirJ belongs to the so-called Radical SAM enzyme family whose members are known to catalyze chemically challenging reactions through radical-based mechanisms [41, 42]. It was also speculated that the NirD, NirL, NirG, and NirH proteins might be responsible for the decarboxylation reaction (a) [38]. 

**Figure 3:**
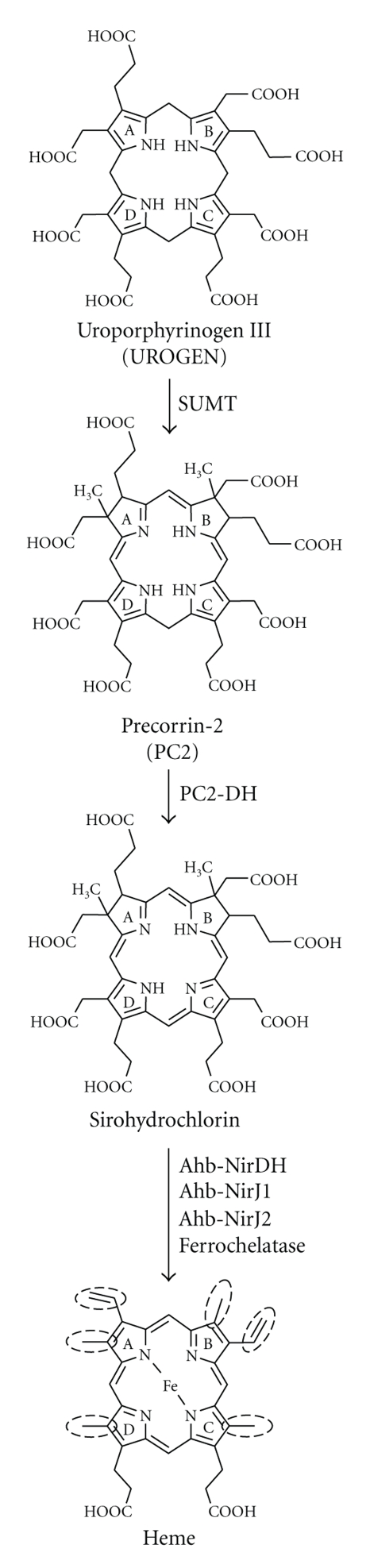
Proposal for the novel, alternative heme biosynthesis pathway in archaea. Archaeal heme biosynthesis starts with the SAM-dependent methylation of UROGEN to precorrin-2 by a SUMT and potentially proceeds *via* oxidation of precorrin-2 to sirohydrochlorin by PC2-DH. The side chain modifications (highlighted by dashed circles) including acetate group decarboxylation on rings C and D, acetate group removal on rings A and B, and vinyl group formation on rings A and B are potentially catalyzed by the Ahb-Nir proteins.

In order to form heme from precorrin-2 the decarboxylation of the acetate groups on rings C and D as in reaction (a) is required and thus might be catalyzed by Ahb-NirD and Ahb-NirH. Further, the acetate side chains on rings A and B have to be removed, probably in a reaction which resembles the mechanism of reaction (b). Consequently, the Radical SAM enzyme Ahb-NirJ2 which shares 38.8% sequence identity with *P. aeruginosa* NirJ is a good candidate for catalyzing this reaction. This function was previously proposed for one of the NirJ-like proteins from *D. vulgaris* [26]. Another reaction which is required for heme formation from precorrin-2 that has, however, no equivalent in heme *d*_1_ biosynthesis is the oxidative decarboxylation of the propionate side chains on rings A and B to the corresponding vinyl groups. This reaction also takes place during the classical heme biosynthesis route in most bacteria and the *Eukaryota*. In bacteria it is catalyzed by either HemF or HemN (see Figure 1(a)). HemN also belongs to the Radical SAM enzyme family [43]. Thus, Ahb-NirJ1 (Radical SAM family member) might catalyze the formation of the required vinyl groups. In summary, we propose that the Ahb-Nir proteins catalyze some of the late reaction steps during archaeal heme biosynthesis from precorrin-2 (Figure 3).

### 3.6. Distribution of the *ahb-nir* Genes over the Archaeal Genomes

In accordance with the proposed function of the *ahb-nir* gene products during the late steps of archaeal heme biosynthesis we failed to detect any of the *ahb-nir* genes in those archaeal genomes without any *hem* genes (Table 1). However, the presence of the *hem* genes in an archaeal genome does not necessarily mean that the *ahb-nir* genes are also present. As mentioned above, out of 47 archaeal genomes containing all five early *hem* genes only 32 also contain the *ahb-nir* genes. The 15 archaeal species which possess the *hem*, but no *ahb-nir* genes probably synthesize their UROGEN solely as precursor for siroheme [44, 45], cobalamin [31] and, in the case of methanogens, for coenzyme F_430_ [46]. However, most likely they do not form heme. Accordingly, almost all of these 15 species also possess both a *SUMT* and a *PC2-DH* gene which are required for siroheme, cobalamin, and coenzyme F_430_ biosynthesis. One exception is *P. torridus* which does not contain a recognizable *PC2-DH* gene. Moreover, *T. acidophilum* and *T. volcanium* do not possess a *SUMT* gene. Interestingly, these three species are the only representatives of the *Archaea* for which *hemE* and *hemH* genes were found (see above). For *P. torridus* and *T. acidophilum* heme-containing proteins were biochemically characterized [18, 20]. However, considering the observation that their genomes lack recognizable *ahb-nir* genes and some of the late *hem* genes their route of heme biosynthesis remains currently unclear.

Within the group of the 32 archaeal species that contain *ahb-nir* genes several subgroups can be recognized. First of all, there are those species for which a complete set of *ahb-nir* genes (*ahb-nirD*, *ahb-nirH*, *ahb-nirJ1*, *ahb-nirJ2*) was found. Out of the 32 genomes containing *ahb-nir* genes 27 contain all four of them. Among the 5 genomes in which not all *ahb-nir* genes are present, those of *Halorhabdus utahensis* and *Methanopyrus kandleri* are missing *ahb-nirD* and *ahb-nirH*. The genomes of *Sulfolobus acidocaldarius*, *Haloquadratum walsbyi*, and *Nirosopumilus maritimus* do not contain *ahb-nirJ1* and *ahb-nirJ2*. Thus, for these five species it is questionable whether they synthesize heme themselves, if at all required. For example, for *M. kandleri* and *N. maritimus* no indications were found in the literature or databases that they possess heme-containing proteins. Further, two subgroups of *ahb-nir* containing archaea can be distinguished depending on whether they possess two distinct *ahb-nirD* and *ahb-nirH* genes or whether they contain an *ahb-nirDH* gene fusion. In fact, almost all archaea possess the fused genes except for those methanogenic archaea which contain the *ahb-nir* genes (Table 1). However, in these methanogens the *ahb-nirD* and *ahb-nirH* genes are always located aside to each other on the genome with the only exception being *Methanosaeta thermophila*. Likewise, the two *ahb-nirJ* genes are also often (15 out of 29) co-localized on the genomes, either as direct neighbors or in close proximity to each other, indicating gene duplication as the origin of the two copies.

Our bioinformatics investigation of 59 archaeal genomes in combination with the experimental evidence that two methyl groups of archaeal heme are derived from *S*-adenosyl-L-methionine strongly suggests that heme biosynthesis in the *Archaea* follows a novel, yet mostly unknown route. It starts with the methylation of UROGEN to precorrin-2 catalyzed by SUMT, followed by the oxidation of precorrin-2 to sirohydrochlorin by PC2-DH and further transformations (decarboxylation of acetate groups, removal of acetate groups, oxidative decarboxylation of propionate to vinyl groups, and insertion of iron) of the macrocycle side chains which are most likely performed by the Ahb-Nir proteins (Figure 3). Clearly, these proposals need to be tested experimentally. Thus, we decided to first verify the predicted functions of the *M. barkeri* proteins Mba_1791 and Mba_1461 as SUMT and PC2-DH, respectively.

### 3.7. Production and Purification of Recombinant Mba_1791 and Mba_1461

**Figure 4:**
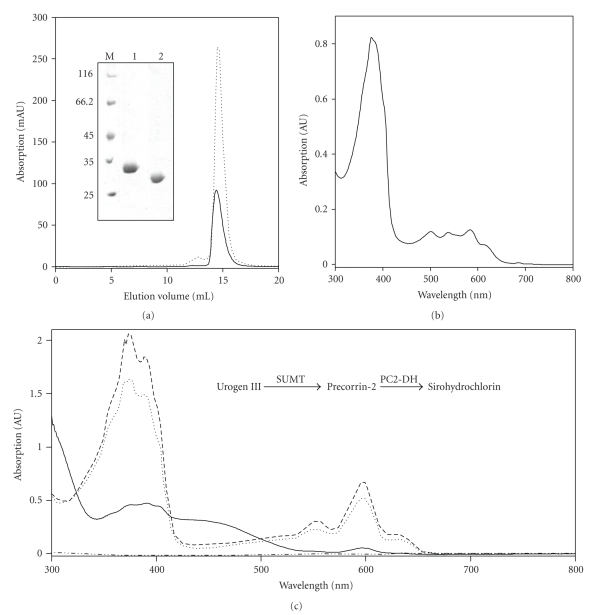
Purification and characterization of Mba_1791 and Mba_1461. (a) SDS-PAGE analysis of purified Mba_1791 (lane 1) and Mba_1461 (lane 2). Gel permeation chromatography revealed native relative molecular masses of 55,300 ± 840 Da for Mba_1791 (dotted line) and 60,800 ± 7,300 Da for Mba_1461 (solid line), respectively. (b) UV-visible absorption spectrum of extracted tetrapyrroles which accumulated during production of recombinant Mba_1791 in *E. coli*. (c) UV-visible absorption spectra of enzyme assays after overnight incubation at 37°C in the anaerobic chamber. Uroporphyrinogen III was produced from ALA by the enzymes HemB, HemC, and HemD (dashed double dotted line). Addition of purified Mba_1791 and SAM to the assay mixture resulted in precorrin-2 formation (solid line). Addition of purified NirE, Mba_1461, and NAD^+^ to the assay resulted in formation of sirohydrochlorin (dotted line). In a coupled enzyme assay containing purified Mba_1791 and Mba_1461 the formation of sirohydrochlorin was also observed (dashed line). For exact details see Section 2.

The *M. barkeri* proteins Mba_1791 and Mba_1461 were recombinantly produced as N-terminal His-tagged fusion proteins in *E. coli*. In both cases the recombinant proteins were produced in a soluble form and in a high yield. We purified Mba_1791 and Mba_1461 to apparent homogeneity using a single affinity-chromatographic step on Ni Sepharose 6 Fast Flow (Figure 4(a)). The purified Mba_1791 exhibited a slight red-brown color. UV-visible absorption spectroscopy suggested the presence of a copurified tetrapyrrole, probably the reaction product of Mba_1791 (data not shown). For other SUMTs (e.g., *P. aeruginosa* NirE) the co-purification of their reaction product has been previously reported [27, 35, 47]. Therefore, the presence of a tetrapyrrole in the purified Mba_1791 was a first hint towards the function of this protein as a SUMT. In contrast, purified Mba_1461 appeared colorless. 

The oligomeric state of Mba_1791 and Mba_1461 was determined by gel permeation chromatography. This experiment revealed a native relative molecular mass of 55,300 ± 840 Da for Mba_1791 and 60,800 ± 7,300 Da for Mba_1461, respectively (Figure 4(a)). The calculated molecular masses based on the amino acid sequences of the proteins are 26,350 Da for Mba_1461 and 27,230 Da for Mba_1791. Thus, gel permeation chromatography suggests a dimeric structure for both proteins. Other SUMTs [48, 49] and PC2-DH [33, 50] are also thought to be dimeric proteins.

### 3.8. Mba_1791 Acts as a SUMT In Vivo

During production of Mba_1791 in *E. coli* a red compound accumulated and remained in the soluble protein fraction of the cell-free extract after disruption of the cells and ultracentrifugation. This compound was extracted using C_18_-reversed phase silica gel and analyzed by UV-visible absorption spectroscopy. The UV-visible absorption spectrum of the extracted compound exhibited an absorption maximum at 378 nm which strongly resembled the previously reported spectra of sirohydrochlorin (Figure 4(b)) [50]. Obviously, recombinantly produced *M. barkeri* Mba_1791 showed significant SUMT activity in the production host *E. coli* which led to the accumulation of sirohydrochlorin, the oxidized form of the SUMT reaction product precorrin-2. Such tetrapyrrole accumulation during recombinant SUMT production has been described before. Depending on the source of the enzyme the formation of either trimethylpyrrocorphin, which is a nonphysiological trimethylated reaction product, or sirohydrochlorin was reported [27, 35, 47, 51–53]. Apparently, Mba_1791 belongs to the class of SUMT enzymes that accumulates sirohydrochlorin and does not catalyze the overmethylation of precorrin-2 to trimethylpyrrocorphin.

### 3.9. *M. barkeri* Mba_1791 Is a SUMT

In order to investigate the *in vitro* activity of Mba_1791 a coupled enzyme assay was performed. The substrate uroporphyrinogen III was produced enzymatically and incubated overnight with recombinant purified Mba_1791. The formation of reaction products was followed using UV-visible absorption spectroscopy (Figure 4(c)). The absorption spectrum of a control assay mixture containing only the uroporphyrinogen III producing enzymes showed no characteristic absorption features under anaerobic conditions. In contrast, the addition of purified Mba_1791 and SAM to the reaction mixture resulted in a yellow colored solution after overnight incubation. The corresponding absorption spectrum exhibited a broad absorption between 350–400 nm and 400–500 nm which is characteristic for precorrin-2 [50, 54]. Consequently, Mba_1791 is indeed a SUMT. Therefore, we will name the enzyme from now on Mba_SUMT. The specific activity of Mba_SUMT was determined with uroporphyrinogen III (produced by chemical reduction of uroporphyrin III) at a concentration of 17 *μ*M, a SAM concentration of 200 *μ*M and a Mba_SUMT concentration of 1.5 *μ*M. Under these conditions we observed a specific activity of 616 nmol precorrin-2 × h^−1^ × mg^−1^ Mba_SUMT corresponding to a turnover of 17 h^−1^. This specific activity is in the same range as the activities observed for other SUMTs [27, 55–57].

### 3.10. *M. barkeri* Mba_1461 is a PC2-Dehydrogenase

In order to verify the postulated PC2-DH activity for Mba_1461 in an *in vitro *assay the enzymatically produced uroporphyrinogen III was converted to the PC2-DH substrate precorrin-2 by addition of the SUMT NirE from *P. aeruginosa*. Additionally, purified *M. barkeri* Mba_1461 and NAD^+^ were added to the reaction mixture. After overnight incubation a UV-visible absorption spectrum of the now purple reaction mixture was measured. The spectrum (Figure 4(c)) corresponds to a typical absorption spectrum of sirohydrochlorin with an absorption maximum at 378 nm [50]. Thus, Mba_1461 exhibited strong PC2-DH activity *in vitro* and can be safely assigned as Mba_PC2-DH.

We also tested the activities of the *M. barkeri* enzymes Mba_SUMT and Mba_PC2-DH in a coupled assay. Both were added to the reaction mixture containing all enzymes for uroporphyrinogen III generation. After overnight incubation the UV-visible absorption spectrum of this assay mixture was similar to the spectrum of the reaction mixture containing *P. aeruginosa* NirE (SUMT) and Mba_PC2-DH showing again the formation of sirohydrochlorin (Figure 4(c)). When SAM and/or NAD^+^ were omitted from this activity assay no formation of sirohydrochlorin was observed (data not shown). 

## 4. Conclusion

In this study we have identified gene clusters in many archaeal genomes that are likely required for the biosynthesis of heme *via* a novel pathway. These gene clusters consist of (i) the known *hem* genes (*hemA^B^*, *hemL*, *hemB*, *hemC*, *hemD*) necessary for the formation of the heme precursor UROGEN, (ii) the *SUMT* and *PC2-DH* genes required for the synthesis of the intermediates precorrin-2 and sirohydrochlorin, and (iii) the *ahb-nir* genes whose protein products are probably responsible for the conversion of sirohydrochlorin into heme (Figure 3). We propose that the detected *ahb-nir* genes are involved in archaeal heme biosynthesis and not in heme *d*_1_ biosynthesis since almost all archaea do not possess a cytochrome *cd*_1_ nitrite reductase. However, we failed to detect any obvious genes encoding potential ferrochelatases within the archaeal heme biosynthesis gene clusters. The *Archaea* do not usually possess a *hemH* gene encoding the bacterial-type ferrochelatase but do contain several copies of genes encoding putative cobalt- and/or magnesium chelatases, although they do not synthesize chlorophylls. These gene products might be involved in the archaeal heme biosynthesis. To confirm our bioinformatics findings and predictions further experimental verification will be required in order to determine the exact function of the Ahb-Nir proteins in the archaeal heme biosynthesis process.

## Figures and Tables

**Table 1:** Heme biosynthesis genes in Archaea.

	Organism	*he* *mA* ^*B*^ ^a^	*hemL*	*hemB*	*hemC*	*hemD*	*SUMT*	*PC2-DH*	*nirD*	*nirH*	*nirJ1*	*nirJ2*	heme^b^	heme *d*_1_	B_12_^c^	siroheme^d^	F_430_	
1	*Aeropyrum pernix* K1	**APE_2296** ^e^	**_2299.1**	**_2300.1**	**_2298.1**	—	_0236.1	***_1491.1***	***_1497.1***	***_1497.1***	*_1655*	*_1652*	+	—	—		—	1
2	*Desulfurococcus kamchatkensis* 1221n	—	—	—	—	—	—	—	—	—	—	—		—			—	2
3	*Ignicoccus hospitalis* KIN4/I	IGNI_0512	**_0683**	_0324	**_0684**	*_0090*	*_0089*	_0483	_0839	_0839	_0397	_0630		—	—		—	3
4	*Staphylothermus marinus* F1	—	—	—	—	—	—	—	—	—	—	—		—	—		—	4
5	*Hyperthermus butylicus* DSM 5456	HBUT_0206	**_0837**	_1386	**_0836**	**_0834**	**_0835**	_0510	*_0036*	*_0036*	*_0035*	*_0037*		—	—		—	5
6	*Metallosphaera sedula* DSM 5348	**MSED_0214**	**_0216**	**_0215**	**_0217**	**_0218**	_0608	**_0213**	_0013	_0013	*_0512*	*_0511*		—	+	+	—	6
7	*Sulfolobus acidocaldarius* DSM 639	**SACI_0777**	**_0779**	**_0778**	**_0780**	**_0781**	_0914	**_0776**	_0711	_0711	—	—	+	—	+	+	—	7
8	*Sulfolobus islandicus* M.14.25	**M1425_1955**	**_1953**	**_1954**	**_1952**	**_1951**	_0226	**_1956**	_1894	_1894	*_1048*	*_1047*		—		+	—	8
9	*Sulfolobus solfataricus* P2	**SSO0180**	**0182**	**0181**	**0183**	**0184**	2435	—	0245	0245	*1631*	*1632*	+	—	+	+	—	9
											*1840*	*1839*						
10	*Sulfolobus tokodaii* 7	**ST0212**	**0215**	**0214**	**0217**	**0218**	0563	**0211**	0293	0293	*0127*	*0126*	+	—	+	+	—	10
11	*Thermofilum pendens* Hrk 5	—	—	—	—	—	—	—	—	—	—	—		—	—	—	—	11
12	*Caldivirga maquilingensis* IC-167	**CMAQ_1730**	_1413	_1740	_1121	*_1901*	_1313	**_1731**	_0150	_0150	*_1900*	_1347		—	—	+	—	12
13	*Pyrobaculum aerophilum* IM2	**PAE0601**	**0594**	**0583**	**0580**	**0589**	**0590**	**0585**	**0581**	**0581**	**0596**	**0579**	+	+	+	+	—	13
14	*Pyrobaculum arsenaticum* DSM 13514	**PARS_2261**	**_2254**	**_2247**	**_2245**	**_2250**	**_2251**	**_2248**	**_2246**	**_2246**	**_2255**	**_2244**	+	+	—	+	—	14
15	*Pyrobaculum calidifontis* JCM 11548	PCAL_2034 _1481	**_1717**	**_1709**	**_1707**	**_1712**	**_1713**	**_1710**	**_1708**	**_1708**	**_1716**	**_1706**	+	+	+	+	—	15
16	*Pyrobaculum islandicum* DSM 4184	PISL_0096	**_0114**	*_0042*	*_0050*	**_0116**	**_0115**	**_0119**	*_0044*	*_0044*	**_0113**	*_0051*		—	—	+	—	16
17	*Thermoproteus neutrophilus* V24Sta	TNEU_1917	**_1900**	*_0957*	*_0968*	**_1898**	**_1899**	**_1896**	*_0962*	*_0962*	**_1901**	*_0969*		—		+	—	17
18	*Archaeoglobus fulgidus* DSM 4304	***AF1975***	**1241**	***1974***	**1242**	0116	**1243**	*1592*	*1593M *	*1594M*	2413	1125	+	—	+	+	—	18
									1594M									
19	*Haloarcula marismortui* ATCC 43049	**RRNAC1708**	2628	2610	*3086*	*3088*	*3087*	**1709**	**1711**	**1711**	3489	1363	+	—	+	+	—	19
20	*Halobacterium sp.* NRC-1	*VNG1774G*	**2326G**	**2322G**	**2330G**	**2332G**	**2331G**	*1775C*	*1776G*	*1776G*	***1185G***	***1184GM***	+	—	+		—	20
21	*Halomicrobium mukohataei* DSM 12286	**HMUK_1612**	_0914	_0925	*_1105*	*_1103*	*_1104*	**_1613**	**_1614**	**_1614**	_1679	_1984	+	—		+	—	21
22	*Haloquadratum walsbyi* DSM 16790:HBSQ001	*HQ3336A*	**3447A**	**3443A**	**3450A**	**3452A**	**3451A**	*3335A*	*3334A*	*3334A*	—	—	+	—	+	+	—	22
23	*Halorhabdus utahensis* DSM 12940	HUTA_2825	_1970	_1362	*_0927*	*_0928*	**_1761**	**_1755**	—	—	_0031	_0734	+	—		+	—	23
24	*Halorubrum lacusprofundi* ATCC 49239	**HLAC_2132**	_2622	_0015	*_2252*	*_2254*	*_2253*	**_2131**	**_2130**	**_2130**	_1215	_2081	+	—	+	+	—	24
25	*Natronomonas pharaonis* DSM 2160	*NP4502A*	1246A	0920A	**1326A**	**1330A**	**1328A**	*4500A*	*4498A*	*4498A*	1546A	1542A	+	—	+	+	—	25
26	*Methanobrevibacter smithii* ATCC 35061	*MSM_0967*	_1233	_1476	_0881	_1504	_1550	*_0968*	—	—	—	—		—	+		+	26
27	*Methanosphaera stadtmanae* DSM 3091	**MSP_1408**	*_1180*	_0416	_1332	*_1191*	*_1192*	**_1407**	—	—	—	—		—	+		+	27
28	*Methanothermobacter thermautotrophicus* delta H^e^	**MTH1012**	228	744	874	*166*	*167*	**1013**	—	—	—	—		—	+	+	+	28
29	*Methanocaldococcus fervens* AG86	MEFER_0496	_1387	_0258	_0770	_1494	_0849	_0978	—	—	—	—		—			+	29
30	*Methanococcus jannaschii* DSM 2661	**MJ0143**	0603	0643	0569	0994	0965	**0140**	—	—	—	—		—	+	+	+	30
31	*Methanocaldococcus vulcanius* M7	*METVU_1458*	_0867	_0542	_0749	_0380	*_1448*	_0492	—	—	—	—		—			+	31
32	*Methanococcus aeolicus* Nankai-3	**MAEO_0052**	_1208	_0916	_1324	_0075	_0861	**_0053**	—	—	—	—		—	+		+	32
33	*Methanococcus maripaludis* S2	**MMP0088**	0224	1258	0872	0394	0966	**0089**	—	—	—	—		—	+		+	33
34	*Methanococcus vannielii* SB	**MEVAN_1100**	_1237	_0571	_0048	_1381	_0289	**_1101**	—	—	—	—		—	+		+	34
35	*Methanocorpusculum labreanum* Z	**MLAB_0523**	**_0525**	**_0524**	**_0526**	_0073	**_0526**	**_0522**	—	—	—	—		—	+		+	35
						_0359												
36	*Methanoculleus marisnigri* JR1	**MEMAR_0980**	**_0982**	**_0981**	**_0983**	_0539	**_0984**	**_0979**	*_0878*	*_0877*	*_0879*	*_0876*		—	+		+	36
37	*Methanospirillum hungatei* JF-1	**MHUN_2562**	**_2560**	**_2561**	**_2559**	_2267	**_2558**	**_2563**	—	—	—	—		—	+		+	37
38	*Methanoregula boonei* 6A8	**MBOO_1238**	**_1235**	**_1236**	**_1234**	_0514	**_1233**	**_1239**	*_0957*	*_0956*	*_0958*	*_0955*		—	+		+	38
39	*Methanosphaerula palustris* E1-9c	**MPAL_1728**	**_1726**	**_1727**	**_1725**	_2749	**_1724**	**_1729**	*_2627*	*_2628*	*_2626*	*_2629*		—			+	39
40	*Methanosaeta thermophila* PT	*MTHE_0049*	**_1126**	*_0050*	**_1125**	**_1124**	**_1124**	—	**_1134**	*_0047*	**_1135**	**_1123**	+	—	+		+	40
41	*Methanococcoides burtonii* DSM 6242	**MBUR_1229**	**_1227**	**_1228**	**_1226**	_1106	_1105	**_1230**	**_1232**	**_1231**	**_1233**	**_1236**	+	—	+	+	+	41
42	*Methanosarcina acetivorans* C2A	**MA0577**	**0581**	**0578**	**0582**	*3034*	*3033*	**0576**	**0574**	**0575**	**0573**	*3035*	+	—	+		+	42
43	*Methanosarcina barkeri* fusaro	**MBAR_A1462**	**_A1464**	**_A1463**	**_A1465**	*_A1792*	*_A1791*	**_A1461**	**_A1459**	**_A1460**	**_A1458**	*_A1793*	+	—	+		+	43
44	*Methanosarcina mazei* Goe1	**MM_1741**	**_1743**	**_1742**	**_1744**	*_0308*	*_0307*	**_1740**	**_1738**	**_1739**	**_1737**	*_0309*	+	—	+		+	44
45	*Methanopyrus kandleri* AV19	*MK0200*	MKT08	*0198*	0746	**1550**	**1548**	1495	—	—	0896	0980		—	+	+	+	45
46	*Pyrococcus abyssi* GE5	—	—	—	—	—	—	—	—	—	—	—		—	—		—	46
47	*Pyrococcus furiosus* DSM 3638	—	—	—	—	—	—	—	—	—	—	—		—	—		—	47
48	*Pyrococcus horikoshii* OT3	—	—	—	—	—	—	—	—	—	—	—		—	—		—	48
49	*Thermococcus gammatolerans* EJ3	—	—	—	—	—	—	—	—	—	—	—		—			—	49
50	*Thermococcus kodakarensis* KOD1	—	—	—	—	—	—	—	—	—	—	—		—	—		—	50
51	*Thermococcus onnurineus* NA1	—	—	—	—	—	—	—	—	—	—	—		—			—	51
52	*Thermococcus sibiricus* MM 739	—	—	—	—	—	—	—	—	—	—	—		—			—	52
53	*Picrophilus torridus* DSM 9790	PTO0918	**0248**	1311	**0249**	**0250**	1435	—	—	—	—	—	+	—	+		—	53
54	*Thermoplasma acidophilum* DSM 1728	TA0536	**0571**	0955	**0572**	**0573**	—	0652	—	—	—	—	+	—	+		—	54
55	*Thermoplasma volcanium* GSS1	TVN0590	**0635**	1100	**0634**	**0633**	—	0924	—	—	—	—		—	+		—	55
56	uncultured methanogenic archaeon RC-I	**RCIX911**	**913**	**912**	**914**	**916**	**915**	**909**	—	—	—	—		—			+	56
57	*Korarchaeum cryptofilum* OPF8	—	—	—	—	—	—	—	—	—	—	—		—			—	57
58	*Nanoarchaeum equitans* Kin4-M	—	—	—	—	—	—	—	—	—	—	—		—	—		—	58
59	*Nitrosopumilus maritimus* SCM1	**NMAR_0510**	*_0490*	**_0509**	*_0491*	*_0493*	*_0492*	**_0511**	**_0512**	**_0512**	—	—		—		+	—	59
60	*Escherichia coli* K-12 MG1655	B1210	0154	0369	**3805**	**3804**	3368	3368	—	—	—	—	+	—	—	+	—	60
61	*Pseudomonas aeruginosa* PAO1	PA4666	3977	5243	*5260*	*5259*	**0510** ^f^	2611	**0515** ^f^	**0512** ^f^	—	**0511** ^f^	+	+	+	+	—	61
62	*Desulfovibrio vulgaris* Hildenborough	***DVU1461***	*3168*	**0856**	1890	0734	0734	***1463***	**0854**	*3167*	**0855**	**0857**	+	—	+	+	—	62

^a^ *he* *mA* ^*B*^ gene encoding glutamyl-tRNA reductase in the listed organisms. In the case of two or more copies for one gene, these copies are only listed if none of them is located in the gene cluster, otherwise only the copy within the cluster is listed. This is valid for all listed genes. Genes colocated within the same gene cluster in a certain species are highlighted in the same font (bold, italics, bold italics).

^b^Organisms possessing heme-containing proteins according to the literature [14–22] are marked with +. For organisms with empty fields the presence of heme-containing proteins was not clearly obvious from literature and bioinformatics data.

^c^Organisms possessing cobalamin biosynthesis genes according to [35] are marked with +. Organisms that do not synthesize cobalamin are marked with — [35]. For organisms with empty fields the ability to synthesize cobalamin was not obvious from the literature.

^d^Organisms possessing siroheme-containing sulfite or nitrite reductases based on sequence homology are marked with +. For organisms with empty fields the presence of siroheme-containing sulfite and nitrite reductases was not clearly obvious from literature and bioinformatics data.

^e^Numbers represent the database gene number. The full database gene numbers including the strain abbreviation is given for the *hemA* ^*B*^ genes. For all other genes the database gene numbers are given without the strain abbreviation. For genes that are located as direct or near neighbors in an individual strain the numbers are written in the same font.

^f^ *P. aeruginosa nirE*, *nirD*, *nirH*, and *nirJ* genes involved in heme *d*_1_ biosynthesis.
